# Nanobody-Functionalized Magnetic Beads for Enrichment of Foodborne Botulinum Toxin Type A Light Chain

**DOI:** 10.4014/jmb.2507.07042

**Published:** 2025-10-13

**Authors:** Hieu Trung Luong, My Bao Kieu, Huong Thi Phi, Nam Hoang Nguyen, Quynh Manh Luu, Cham Thi Vu, Dat Thanh Pham, Yen Bao Pham, Loan Thi Hong Nguyen, Trung Thanh Nguyen, Nguyet Thi Anh Nguyen, Toan Van Trinh, Thuy-Anh Thi Tran, Nhung Thi Hong Le

**Affiliations:** 1 Faculty of Biology, VNU University of Science, Hanoi, Vietnam, 334 Nguyen Trai, Thanh Xuan, Ha Noi 10000, Vietnam; 2 The Faculty of Physics, VNU University of Science, Hanoi, Vietnam, 334 Nguyen Trai, Thanh Xuan, Ha Noi 10000, Vietnam; 3 Key Laboratory of Enzyme and Protein Technology, VNU University of Science, Hanoi, Vietnam, 334 Nguyen Trai, Thanh Xuan, Ha Noi 10000, Vietnam; 4 National Institute for Food Control, 65 Pham Than Duat, Mai Dich, Cau Giay, Ha Noi 100000, Vietnam; 5 Research Institute of Stem Cell and Gene Technology, College of Health Sciences, VinUniversity, Vinhomes Ocean Park, Gia Lam, Ha Noi 1310, Vietnam; 6 Institute of Tropical Medicine, Joint Vietnam-Russia Tropical Science and Technology Research Center, Ha Noi 10000, Vietnam

**Keywords:** Nanobody, botulinum toxin, light chain, magnetic beads, enrichment, botulinum serotype A

## Abstract

In this study, for the first time, nanobody conjugated beads were developed to enrich botulinum serotype A. We evaluated two types of nanobody A8-functionalized magnetic beads, both of which effectively captured and enriched BoNT/A from dilute solutions and complex protein mixtures, achieving a concentration increase of approximately 12- to 30-fold. The A8@N-beads showed higher binding capacity than the A8@S-beads, suggesting that our self-synthesized NH_2_ beads work well in this application. Our data serve as evidence that using different bead types with nanobody conjugation is a promising enrichment approach for further detection of Lc/A, particularly in applications involving low-abundance targets or complex sample matrices, such as food safety screening or clinical diagnostics.

## Introduction

Botulinum neurotoxins (BoNTs) are a toxin family comprising seven major serotypes (designated A to G) produced by the bacterium *Clostridium botulinum* [1]. These toxins target motor neurons to block the release of neurotransmitters, causing botulism, which is characterized by flaccid paralysis (loss of muscle contraction ability) [2]. The toxin (holotoxin) consists of two domains: i) the 50 kDa light chain (Lc) is the zinc-containing active site, and ii) the 100 kDa heavy chain (Hc). The Hc domain facilitates the endocytosis of the toxin into the neuronal cell, where the Lc domain, a protease, then cleaves specific N-ethylmaleimide-sensitive factor attachment receptor (SNARE) proteins, thereby inhibiting neurotransmitter release (reviewed in [3]). Among the seven serotypes, BoNT serotype A (BoNT/A) is responsible for the majority of human botulism cases [4]. The high toxicity of BoNT/A and botulinum toxin in general raises explicitly significant public health and economic concerns. However, detecting this toxin in food is challenging due to its low concentration and the complexity of the food matrix. Concentrating BoNT/A from food samples using conjugated magnetic beads before detection might enhance the limit of detection and reduce the inhibitory effects of the food matrix.

Magnetic bead-based enrichment has become widely used in biological applications, and some bead types can be used to separate antigens from the matrix. These bead types include agarose beads [5], and magnetic beads, such as those coated with streptavidin [6, 7] or NH_2_/COOH [8], or those functionalized with a thiol group [8]. Different bead types can have varying enrichment efficiencies and may impact downstream detection methods. Here, we evaluated two magnetic bead types to enrich the recombinant Lc/A: self-synthesized NH_2_-coated beads (N-beads) and commercial streptavidin-coated beads (S-beads). We then conjugated them with nanobody A8 to form two functionalized bead types, referred to respectively as A8@N-beads and A8@S-beads. Furthermore, we sought in this analysis to highlight the possibility of integrating the magnetic-bead system into the detection downstream.

Nanobodies (also known as single-domain antibodies) are antigen-binding domains derived from the heavy-chain antibodies that are found in camelids and sharks. In recent years, nanobodies have gained increased interest as alternatives to antibodies due to their small size (13-15 kDa), stability, and ease of modification when produced using recombinant technology [9-12]. In addition to their many other applications, nanobodies have been utilized for antigen enrichment and detection [7, 13, 14]. For botulinum, Harmsen *et al*., synthesized high-affinity nanobody multimers targeting BoNT/C and BoNT/D. They incorporated them into a magnetic bead-based enrichment system followed by mass spectrometry analysis, achieving a detection sensitivity of 13 pg/ml for BoNT/D. However, no published research has yet explored the use of nanobodies for enriching botulinum toxin serotype A.

In this study, we demonstrated the potential of nanobody-based enrichment for botulinum serotype A. Among several promising nanobodies, the nanobody Alc-B8 (called nanobody A8) has been shown to have strong binding affinity to the light chain of BoNT/A [15, 16], as well as potent protease-inhibiting activity against the protease function of Lc/A [15, 17, 18]. Additionally, we report for the first time the application of nanobody A8 for the selective enrichment of BoNT/A light chain (Lc/A).

## Materials and Methods

### Chemicals and Reagents

His-Tag Monoclonal Antibody (HRP-66005-1-Ig), Goat Anti-Mouse IgG AP (SA00002-1), and Anti-Mouse HRP (SA00001-1) were obtained from Prointech (USA). *Clostridium botulinum* BoNT-A Light Chain Polyclonal Antibody was purchased from R&D Systems (USA), and Goat Anti-Llama IgG H&L (HRP, Ab112786) was obtained from Abcam (UK). An EZ-Link Sulfo-NHS-LC-Biotinylation Kit and Dynabeads MyOne Streptavidin T1 were obtained from Thermo Fisher Scientific (USA), and GangNam-STAIN Prestained Protein Ladder was purchased from iNtRON (Republic of Korea).

### Plasmid Constructs

Plasmid pBN3 was used to express the coding sequence of the light chain of botulinum neurotoxin type A with a C-terminal 6xHis tag (Addgene, #251561, USA). SNAP25 in the pET28a expression vector was codon-optimized with a C-terminal 6xHis-tag (Addgene, #168031). The gene encoding nanobody A8 (GenBank No. FJ643070.1) was also synthesized and codon-optimized by GenScript. The cDNA was cloned into the pET28a vector downstream of a thioredoxin (Trx) tag with 6-histidine tags at both the N- and C-termini. Two thrombin cleavage sites were introduced. The gene encoding Lc/B was synthesized and codon-optimized by NovoPro Bioscience (China).

### Protein Expression and Purification

Plasmids were transformed into *Escherichia coli*. Transformed bacteria were grown in Luria-Bertani (LB) agar medium in the presence of chloramphenicol, kanamycin for A8 [18, 19], and similar conditions for SNAP25 and chloramphenicol, as well as ampicillin for Lc/A. Transformations were confirmed by colony PCR using T7 primer. Positive colonies were picked and cultured in LB medium with kanamycin (50 μg/ml), ampicillin (100 μg/ml), and chloramphenicol (34 μg/ml) at 37°C under vigorous shaking. Protein expression was induced with different isopropyl-β-D-thiogalactopyranoside (IPTG) concentrations when the OD_600_ reached 0.6. The culture was then incubated at 16°C for 16 h. Following that, cells were harvested by centrifugation at 6,000 ×*g* and 4°C for 10 min, and then stored at -80°C.

Next, the cells were disrupted by sonication (15 cycles of 30 sec) in the lysis buffer (20 mM Tris-HCl, 500 mM NaCl, 10% glycerol, 20 mM imidazole, and 1 mM phenylmethylsulfonyl fluoride (PMSF). The lysate was centrifuged at 10,000 ×*g* for 10 min at 4°C. The supernatant was collected for purification using a His-tag affinity Ni-NTA agarose column, which was equilibrated with an equilibration buffer before loading of the supernatant. After incubation for 30 min, the column was washed with a washing buffer to remove non-specifically bound proteins. Target proteins were then eluted using an elution buffer containing 250 mM imidazole. The concentration of protein was measured by UV absorbance at 280 nm, and protein fractions were analyzed via SDS-PAGE and western blotting. For further purification, the proteins were desalted using a PD-10 desalting column and stored in a storage buffer containing 20 mM Tris-HCl, 150 mM NaCl, and 10% glycerol.

### Western Blotting Using Nanobody A8 to Detect Recombinant Lc/A

The nitrocellulose membrane was incubated in 10% ethanol for 1 min before being air-dried. A total of 1 μg for each lane of Lc/A was mixed with loading dye buffer and loaded onto SDS-PAGE gels, which were then separated using a 12% SDS-PAGE gel. Proteins were then transferred onto a nitrocellulose membrane, which was blocked with PBS containing 5% skim milk.

Whenever nanobody A8 was used as the primary antibody, the membrane was subsequently incubated with nanobody (1:10) for 1 h, followed by incubation with the secondary anti-llama antibody conjugated with HRP for an additional hour. After each incubation step, the membrane was washed three times with PBS containing 0.05%Tween 20. TMB substrate was used for visualization.

For commercial anti-Lc/A antibody as the primary antibody, a concentration of 0.2 μg/ml was used, and the secondary antibody concentration was 0.067 μg/ml. The substrate BCIP/NBT was used for visualization.

### Enzyme-Linked Immunosorbent Assay (ELISA)

A titrating nanobody was also used. First, ELISA plates were coated with 100 μl of Lc/A at a concentration of 5 μg/ml/well. The titrating nanobody assay was initiated using a 40 μg/ml concentration of nanobody A8, followed by serial 1:2 dilutions before the detection step. The experiments were performed in triplicate. For titration of antigen Lc/A, the ELISA plates were coated with serial dilutions (1:2) of Lc/A at 100 μl/well, and the plates were blocked with 5% skim milk in PBS. Nanobody A8 was prepared at a concentration of 10 μg/ml in PBS, and added to the wells, where it was incubated for 1 h at RT. This was followed by incubation with HRP-conjugated anti-llama antibody at a concentration of 0.17 μg/ml. The wells were also washed with PBS containing 0.005%Tween 20 after each incubation step. For commercial anti-Lc/A antibody as the primary antibody, 1.6 μg/ml of anti-Lc/A was used, and for the secondary antibody, anti-mouse HRP was used at a concentration 0.067 μg/ml. Nanobody/antibody binding was detected using TMB substrate.

### Lc/A Protease Inhibitor Assay

An Lc/A protease assay was employed along with SNAP25 substrate to identify nanobody A8 inhibition of Lc/A activity. SNAP25 contains a 6xHis-tag and YFP at the C-terminus, joined by a SNAP25 linker sequence. The substrate was cleaved by Lc/A at the SNAP25 sequence, and the cleavage product consisted of two proteins with sizes of 33.9 kDa and 30.2 kDa. The inhibition assay used various A8:Lc/A molar ratios. The results were assessed using SDS-PAGE. Inhibitory efficiency was analyzed with ImageJ. The percentage of cleavage was calculated based on the band intensity of the uncleaved substrate relative to that of the input substrate.

### Functionalized Magnetic Beads

For streptavidin beads (S-beads), nanobody A8 was biotinylated using a Sulfo-NHS-LC-Biotinylation Kit and purified on desalting columns. Biotinylation was confirmed through SDS-PAGE, western blot, and dot blot analysis. Immunoprecipitation was performed using biotinylated nanobody A8 coupled to Dynabeads MyOne Streptavidin T1 (Thermo Fisher Scientific). The magnetic beads (0.3 mg) were first washed three times with PBS. Then, 4 μg of biotinylated nanobody A8 was incubated with magnetic S-beads in a volume of 150 μl at RT for 30 min with gentle rotation, after which the unbound nanobodies were washed away with PBS containing 0.01%Tween 20.

For N-beads, 20 μg of nanobody A8 was immobilized onto 0.3 mg of beads in a volume of 150 μl via covalent amide bond formation, facilitated by 100 μg of 1-ethyl-3-(3-dimethylaminopropyl) carbodiimide (EDC) and 60 μg of N-hydroxysuccinimide (NHS) in PBS at pH 6.0 for 1 h at RT. Excess and unbound nanobody was removed by washing the beads with PBS (pH 7.4) supplemented with 0.01% (v/v) Tween 20 (wash buffer).

### Capturing Lc/A by Functionalized Beads

The nanobody-functionalized beads were subsequently blocked with 0.05% (w/v) bovine serum albumin (BSA) for 30 min at RT to prevent non-specific binding. After blocking, the beads were washed three times with wash buffer, then incubated with Lc/A at RT for 1-2 h. Following incubation, the beads were subjected to three additional wash steps with wash buffer to remove nonspecifically bound proteins. The addition of SDS loading buffer eluted Lc/A from the beads, and SDS-PAGE analysis was performed on the eluates. Protein band intensity was quantified by densitometry using ImageJ. For the bead capacity analysis, the samples were concentrated with SpeedVac Vacuum Concentrators (Thermo Fisher Scientific, model no. SPD1010-230) at RT, using a pressure of 0.51 for 2 h. For the specificity experiment, *E. coli* crude protein extracts overexpressing Lc/A, with or without the addition of 4 μg of purified Lc/B, were analyzed.

### Image Analysis

Protein band intensities on SDS-PAGE gels were quantified using the Fiji ImageJ software. Statistical analyses and graph plotting were performed using GraphPad Prism (Version 8.0.2).

The inhibitory activity of nanobody A8 against the protease Lc/A was calculated using the following formula:

Inhibition (%) = (Input band intensity – Uncleaved band intensity) / Input band intensity × 100.

Quantitative densitometry for the binding capacity and enrichment efficiency of Lc/A by functionalized beads was analyzed using the formulas below:

Binding amount = Input amount – (Unbound band intensity / Input band intensity) × Input amount;

Recovery amount = Input amount – (Eluted band intensity / Input band intensity) × Input amount.

The Input amount was determined by measuring absorbance at A280.

### Data Analysis

The inhibitory Kd (dissociation constant) was estimated on GraphPad Prism (Version 8.0.2) using non-linear regression with one-site specific binding.

## Results

### Nanobody A8 Selectively Captures and Inhibits Lc/A

**Fig. 1:**
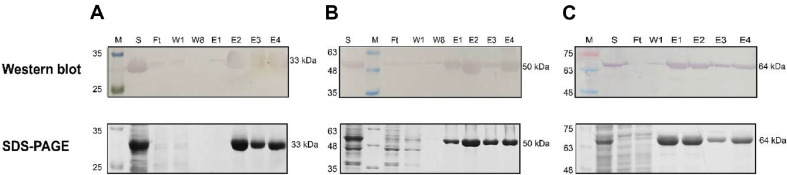
Recombinant synthesis of nanobody A8 and recombinant light chain botulinum neurotoxin type A. Analysis of purified nanobody A8 (**A**) Lc/A (**B**) and SNAP25 (**C**) by western blotting and SDS-PAGE. Detection by western blotting used Mouse Anti-6xHis-tag as primary antibody and Goat Anti-Mouse-AP as secondary antibody. M: Protein ladder; S: supernatant; T: total; P: pellet, FT: Flow-through; W: Wash with 25 mM imidazole; E: Elute obtained with 250 mM imidazole buffer.

To prepare the protein materials, three recombinant proteins, nanobody A8, LcA, and SNAP25, were expressed in *E. coli* and purified using affinity chromatography. Purified A8 (33 kDa) (Fig. 1A), Lc/A (50 kDa) (Fig. 1B), and SNAP25 (64 kDa) (Fig. 1C) were analyzed by SDS-PAGE and western blotting, showing that all three proteins were efficiently purified, with protein yields of 15 mg, 7 mg, and 25 mg, respectively, per liter of culture.

**Fig. 2:**
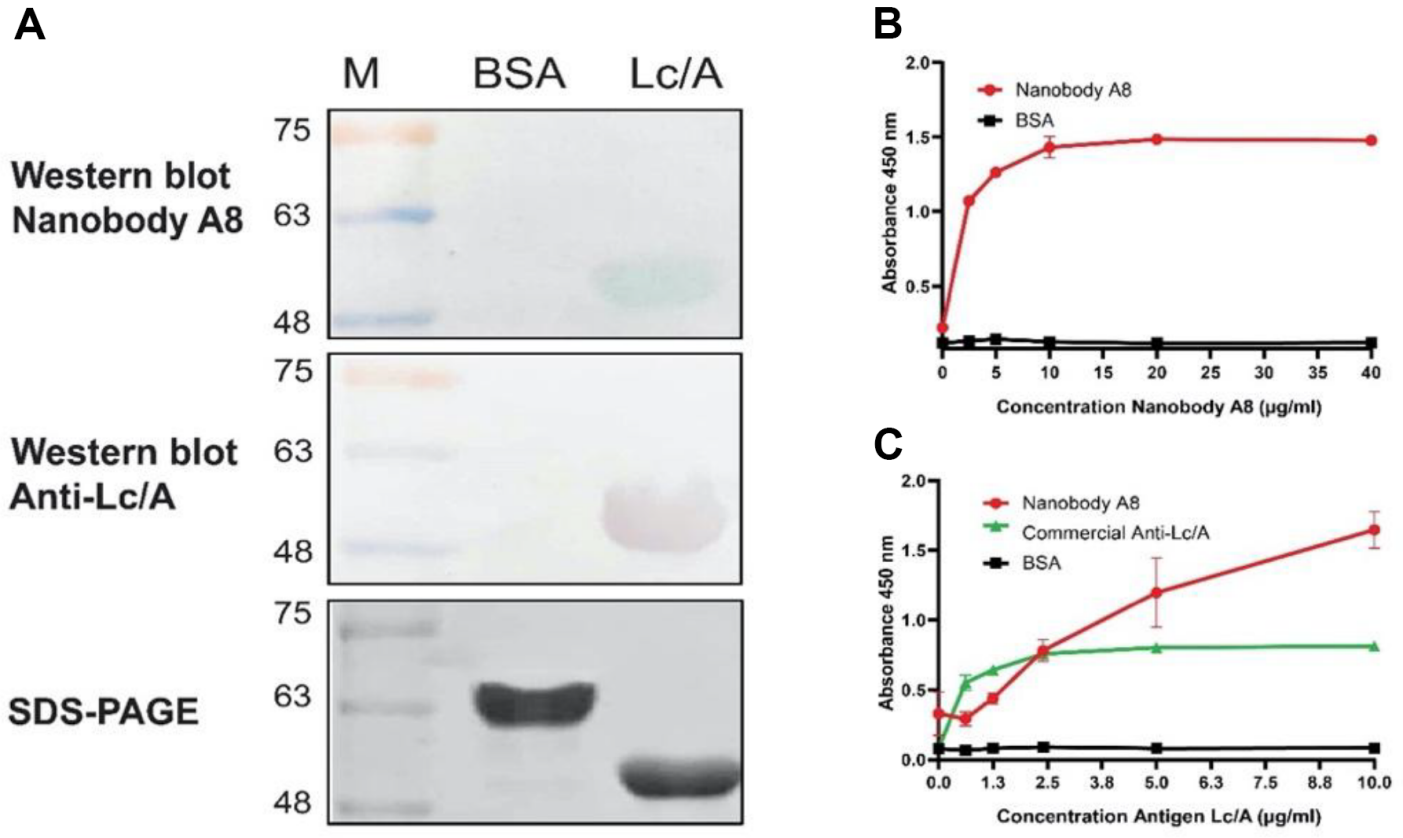
Characterization of nanobody A8 binding and inhibitory activity. Western blot and SDS-PAGE analysis and detection of Lc/A used nanobody A8 as primary antibody and commercial antibody (Anti-Lc/A) as positive control (**A**). ELISA assay evaluating binding of nanobody A8 and Lc/A, (**B**) antibody titration and antigen titration (**C**). M: Protein ladder; BSA (Bovine serum albumin), Lc/A: purify Lc/A. Data from triplicated experiments.

To evaluate the ability of nanobody A8 to detect Lc/A, A8 was used as the primary antibody at a concentration of 100 μg/ml, followed by detection with a secondary anti-llama antibody in western blotting. The results demonstrated specific binding of A8 to Lc/A, as indicated by the presence of a distinct band at ~50 kDa. No signal was observed for the negative control, protein BSA (Fig. 2A). Similarly, a commercial anti-Lc/A antibody was used as the primary antibody, followed by the corresponding secondary antibody, and also recognized Lc/A and showed no binding to BSA.

In the ELISA assay, a nanobody titration was performed to determine the optimal concentration of nanobody A8 for detecting 0.5 μg of Lc/A. The results showed that a minimum concentration of 10 μg/ml of nanobody A8 was required to achieve a strong signal (Fig. 2B). Subsequently, Lc/A antigen titration was conducted with the optimized concentration of 10 μg/ml for nanobody A8 and the recommended concentration for the commercial anti-Lc/A antibody. The lowest detectable concentration of Lc/A was found to be 1.25 μg/ml for both nanobody A8 and the commercial antibody (Fig. 2C). The binding affinity of nanobody A8 to Lc/A was estimated to have a Kd of 33 nM, which is consistent with a previous report describing nanobody affinities in the low nanomolar range [15]. Notably, at higher antigen concentrations of Lc/A, nanobody A8 (at 10 μg/ml) generated even stronger signals than the commercial anti-Lc/A antibody (at 1.6 μg/ml). This enhanced signal might be attributed to the higher concentration of nanobody (10 μg/ml) than that of the commercial anti-Lc/A antibody (1.6 μg/ml). A higher concentration of commercial anti-Lc/A antibody should be tested for better comparison. However, the smaller molecular size of nanobody A8 should also be taken into consideration, as it allows for a higher surface density and more efficient epitope accessibility compared with conventional antibodies.

**Fig. 3:**
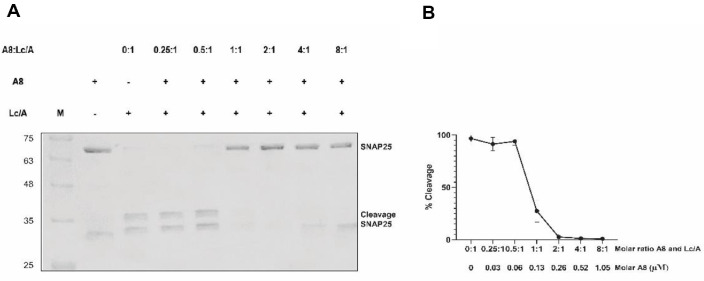
Inhibitory activity of nanobody A8 against the protease Lc/A. SDS-PAGE-based substrate cleavage assays (**A**). Percentages of substrate cleavage analyzed using ImageJ. Data from triplicated experiments (**B**).

The inhibitory effect of nanobody A8 on the enzymatic activity of LcA was evaluated at varying molar ratios of A8 to Lc/A. SDS-PAGE analysis revealed that nanobody A8 completely inhibited the cleavage of SNAP-25 by Lc/A at A8:Lc/A molar ratios greater than 2:1 (Fig. 3A). Notably, inhibition was already detectable on SDS-PAGE at a 1:1 molar ratio, indicating the potent activity of nanobody A8. The half-maximal inhibitory concentration (IC_50_) of nanobody A8 was estimated using GraphPad Prism to be approximately 104 nM at 0.8 mole nanobody/1 mole Lc/A (Fig. 3B). These results demonstrate that nanobody A8 effectively inhibits the SNAP-25–cleaving activity of Lc/A, consistent with previous findings [20].

### Enrichment of Lc/A Using Nanobody A8-Conjugated Magnetic Beads

Antibody-conjugated magnetic beads are widely used to concentrate antigens from diluted, large-volume samples, and to separate target antigens from other contaminants present in diagnostic matrices. This process is referred to as an enrichment step. In this study, we employed two types of magnetic beads: i) streptavidin-coated beads (S-beads) and ii) amine-functionalized (NH_2_-coated) beads (N-beads), to conjugate with nanobody A8 for the enrichment of Lc/A.

**Fig. 4:**
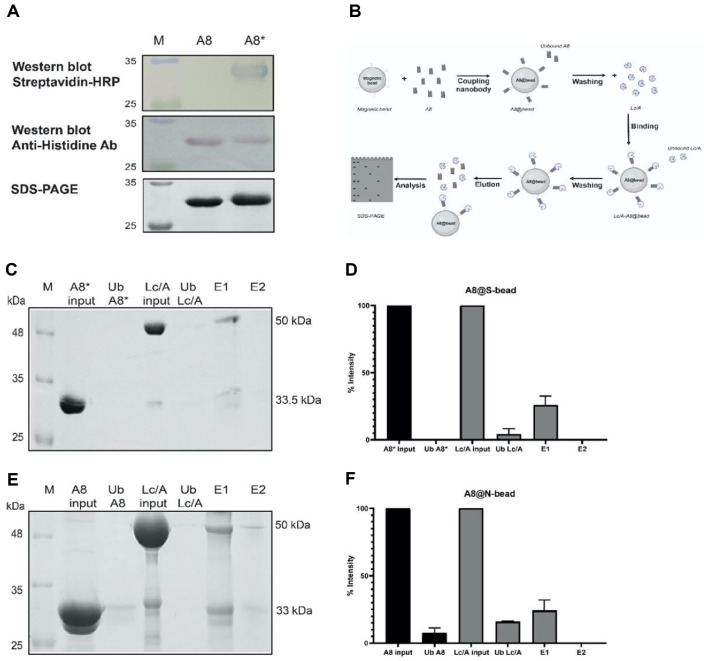
Binding capacity of two bead types to A8 and Lc/A. SDS-PAGE, western blot of SDS-PAGE gel analysis, and western blotting using Anti-6xHistidines and Streptavidin-HRP to detect biotinylated nanobody A8 (**A**). Diagram of the immunoprecipitation process of S-beads or N-beads (**B**); analysis on SDS-PAGE of S-beads (**C**); and protein quantification using ImageJ of S-beads (**D**); analysis on SDS-PAGE of N-beads (**E**); and protein quantification using ImageJ of N-beads (**F**). M: Protein ladder; A8: nanobody A8, A8*: biotinylated A8; Ub: Unbound; Lc/A: purify Lc/A; E: Elute with 2x loading sample buffer.

Regarding the S-bead, we first biotinylated the nanobody A8 and verified it using SDS-PAGE and western blot. Compared to the unmodified nanobody A8 (33 kDa), biotinylated nanobody A8 (A8*) migrated more slowly on SDS-PAGE, appearing as a higher molecular weight band (~33.5 kDa) (Fig. 4A). The biotinylation on nanobody A8 was further confirmed using HRP-conjugated streptavidin in a western blot. The shift in electrophoretic mobility of nanobody A8 before and after biotinylation is likely due to the altered charge distribution resulting from the addition of biotin. The nanobody A8 was then incubated with S-beads, and the supernatant obtained after collecting the beads with a magnet was referred to as the unbound fraction, as illustrated in Fig. 4B. The analysis on SDS-PAGE showed that the A8* was no longer detectable in the unbound fraction, indicating that nearly all of the input A8* had bound to the streptavidin-coated beads, forming functional A8-conjugated beads (A8@S-beads) (Fig. 4C). Using ImageJ analysis, we estimated that 0.3 mg (equivalent to 30 μl) of streptavidin-coated beads could immobilize up to 4 μg of biotinylated A8 (Fig. 4D). These A8@S-beads were then used to capture purified recombinant Lc/A. The unbound Lc/A was collected, and SDS-PAGE analysis revealed only a faint band (~50 kDa) in the unbound fraction (Fig. 4C). Quantitative densitometry using ImageJ indicated that approximately 95% of the input Lc/A (equivalent to 2.7 μg) was successfully captured by 0.3 mg of A8@S-beads. To elute bound Lc/A, the beads were heated at 95°C for 5 min in SDS loading buffer containing DTT. This treatment was intended to disrupt the interaction between Lc/A and nanobody A8*, and may have also released a portion of A8* from the beads. Approximately 30% of the bound Lc/A (~1 μg) was recovered in the eluted fraction (Fig. 4D).

Similarly, nanobody A8 was covalently conjugated to magnetic beads containing NH_2_ groups through peptide bond formation catalyzed by EDC and NHS, resulting in A8@N-bead (Fig. 4E and 4F). The data showed that 0.3 mg of N-beads could bind approximately 18 μg of nanobody A8, which subsequently enables the capture of 9.5 μg of Lc/A. Of the bound Lc/A, approximately 30% (3.3 μg) was recovered in the eluted fraction (Fig. 4F).

Compared to S-beads, N-beads demonstrated 4–5-fold higher loading capacity for nanobody A8, and nearly 10-fold higher capacity for Lc/A. This difference may be attributed to the smaller size of NH_2_-beads (0.7 μm) compared to S-beads (1 μm), as well as differences in surface functional group density (2–3 × 10^5^ streptavidin molecules per S-bead vs. 8–9 × 10^6^ NH_2_ groups per N-bead.

**Fig. 5:**
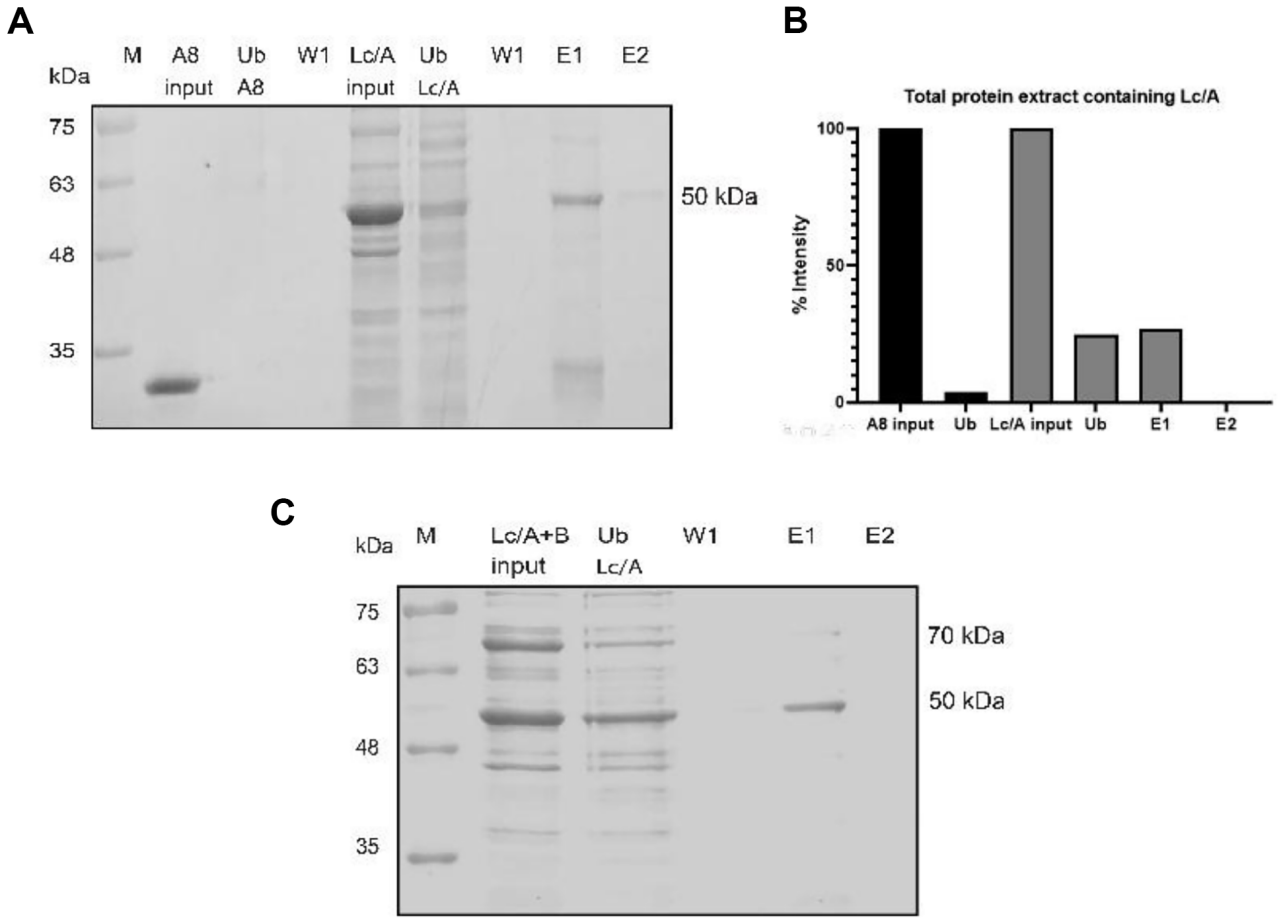
Specificity of A8@N-beads to Lc/A. Purification of Lc/A from *E. coli* crude protein extract overexpressing Lc/A using A8@N-beads on SDS-PAGE (**A**) band intensities were quantified using ImageJ (**B**). Purification of Lc/A from *E. coli* crude protein extract overexpressing Lc/A with Lc/B addition using A8@N-beads on SDS-PAGE (**C**). M: Protein ladder; A8: Nanobody A8; FT: Flow-through; S Lc/A: Total protein extract containing Lc/A; W: Wash with washing buffer; Lc/A: purify Lc/ A; E: Elution with 2x SDS loading sample buffer.

The specific purification capability of A8@N-bead for Lc/A was further evaluated using a crude protein extract containing Lc/A (Fig. 5A and 5B), and the crude extract containing Lc/A spiked with Lc/B (70 kDa) (Fig. 5C). SDS-PAGE analysis showed that Lc/A (~50 kDa) was specifically purified from the protein mixture, even in the presence of Lc/B. This confirms the specificity of the developed beads and demonstrates its applicability to real diagnostic scenarios. The faint band at 33 kDa corresponded to the partially liberated A8 (Fig. 5A).

**Fig. 6:**
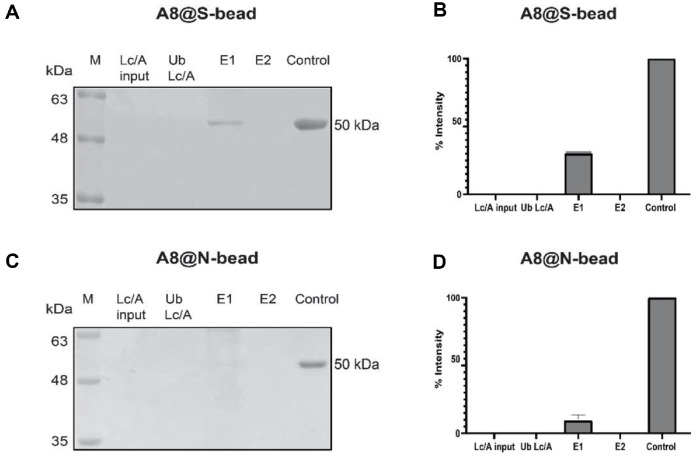
Enrichment of Lc/A using two magnetic beads. Enrichment efficiency using A8@S-beads complexes (**A**) and A8@N-beads complexes (**C**) was analyzed by SDS–PAGE, and band intensities were quantified of A8@S-bead (**B**) and A8@Nbead (**D**) complexes using ImageJ. M: Protein ladder; Lc/A input: purify Lc/A; Ub: Unbound; W: Wash with washing buffer; E: Elute with 2x loading sample buffer; control: A control band of Lc/A with a defined input amount.

For the enrichment assay, an input sample containing Lc/A at 1 ng/μl (500 ng in 500 μl) was incubated with 0.1 mg of A8@S-beads or A8@N-beads. As shown in the SDS-PAGE results, the low concentration of input Lc/A did not show a visible band, whereas a distinct Lc/A band was observed in the eluted fraction (E1) (Fig. 6A and 6C). Based on comparison with the control bands of known concentrations, the eluted Lc/A was estimated to be concentrated to 30 ng/μl in a 10 μl volume (300 ng total) using A8@S-beads, and 12 ng/μl in a 10 μl volume (120 ng total) using A8@N-beads (Fig. 6B and 6D). This corresponded to 30-fold and 12-fold enrichment, respectively. Approximately 60% and 24% of the total Lc/A were recovered from the beads. Optimizing the elution buffer may further enhance recovery efficiency and yield better enrichment efficiency.

Overall, these results confirm the effective enrichment capability of both A8@S-beads and A8@N-beads. These findings highlight the potential of nanobody A8 immobilized on magnetic beads for separating and concentrating Lc/A from diluted or complex food matrices in detection assays.

## Discussion

In this study, nanobody A8 was demonstrated to be suitable for coating onto magnetic bead systems, enabling effective enrichment of Lc/A. The enrichment strategy serves two key purposes: (i) to isolate the toxin from other components in complex matrices, such as food or clinical samples, and (ii) to increase the concentration of diluted toxin, thereby improving detection sensitivity. In our case, the enrichment increased Lc/A concentration by 12- to 30-fold. This highlights the potential of nanobodies in bead-based diagnostic workflows, and also in other areas, such as biosensors. Similar bead-based systems have been reported to improve other target enrichment by up to 25-fold [21], or even 300-fold [22], and to enhance detection sensitivity by up to three orders of magnitude (10³) when combined with analytical techniques, such as mass spectrometry [23].

Here, we demonstrate for the first time the use of a nanobody for the enrichment of botulinum toxin type A. Previous studies on enrichment of serotype A have relied on antibodies or peptides [6, 8]. Due to their small size, nanobodies may lose activity upon bead immobilization depending on their orientation; however, in our case, efficient immobilization was achieved. We confirmed the enrichment capability of nanobody A8 for Lc/A using two types of magnetic beads: streptavidin-coated and NH_2_-functionalized beads. Both bead types demonstrated efficient binding and enrichment of Lc/A from dilute and complex protein mixtures, achieving concentration factors ranging from 12- to 30-fold. Improving the elution buffer can even enhance the concentration. Among the two bead types, A8@N-beads showed a higher binding capacity. This may be due to the smaller size and higher functional group density on NH_2_-beads compared to streptavidin-beads. The recovery of Lc/A from the beads was better with A8@S-bead by 8-fold. The N-bead somehow provides better accessibility and surface presentation of Lc/A for elution. However, further validation is needed to confirm this. Optimization of the buffer conditions—such as adjusting the pH for metal ions or modifying the salt concentration—may be required depending on the application. In some cases, however, it is not necessary to release the antigen from the beads, as a bead-based detection system can be employed directly.

Magnetic bead-based separation offers rapid, scalable, and centrifuge-free sample preparation. This approach also addresses the limitation that arises when the bacteria are no longer viable, but the toxin is still present in the food sample. Moreover, in a diagnostic context, rapid detection is critical because the effective treatment window is limited to approximately 36 h. Indeed, our enrichment process took 3-4 h compared to other enrichment methods for botulinum toxin, such as culture enrichment, which might take 3-5 days ([24]; AOAC Official Method 977.26). Enrichment thus reduces overall detection time. In the same line, synaptotagmin II peptide-functionalized beads enabled the detection of 16.6 pg of BoNT/B in whole milk within 1 h [8]. Similarly, bead-based enrichment followed by FRET detection increased the Lc/A conversion enzymatic rate by 18-fold. In this approach, BoNT/A immobilized on beads exhibited improved substrate binding affinity, achieving detection within 2–3 h [25]. In another study, antibody-conjugated beads were shown to concentrate sample volumes of 100–500 μl down to 20 μl, achieving an limit of detection (LOD) in human serum of 200–400 pg/ml [26]. Using a similar bead-based enrichment approach, the LOD for botulinum toxoid was reported as 16 ng/ml or 320 pg [6]. This enrichment strategy is also applicable to other targets, including *E. coli* and *B. globigii*, where it enabled a 30-min detection time and improved the LOD by 100-fold [27].

Regarding downstream detection, both endopeptidase-based and immuno-based methods can be applied in general. Some of the detection methods are endopeptidase assays combined with mass spectrometry (endopep-MS) for highly specific detection [26], fluorescence resonance energy transfer (FRET)-based assays [25], and immunofluorescence-based methods [8]. In our case, nanobody A8 not only binds to Lc/A, but also inhibits its enzymatic activity. Therefore, immuno-based detection methods may be more suitable, especially when paired with another peptide or antibody that recognizes a different epitope of Lc/A, enabling sandwich-format detection. If downstream analysis requires assessment of Lc/A enzymatic activity, the enriched toxin must first be eluted from the bead system. In this scenario, the recovery rate of enzymatically active Lc/A will depend on the efficiency of the elution buffer. For such applications, methods like endopep-MS could be employed following elution. In conclusion, our results highlight the utility of nanobody-based magnetic beads for Lc/A enrichment, with broad implications for potential diagnostic applications.
